# Impact of a hybrid flipped classroom and case-based learning model on learning outcomes and competency development in *Epidemiology*

**DOI:** 10.3389/fmed.2026.1858834

**Published:** 2026-07-15

**Authors:** Junqiang Wang, Yeying Wang, Zexing Yang, Ling Zhao, Xiaoshuang Xu, Xuemin Feng, Ying Chen, Limei He

**Affiliations:** 1 Department of Epidemiology and Health Statistics, School of Public Health, Kunming Medical University, Kunming, China; 2 Department of Reproductive Genetics, The First Affiliated Hospital of Kunming Medical University, Kunming, China; 3 Department of Endocrinology and Metabolism, The Second Affiliated Hospital of Kunming Medical University, Kunming, China; 4 Yunnan Infectious Diseases Hospital, Kunming, China

**Keywords:** case-based learning, *Epidemiology* education, flipped classroom, hybrid teaching model, learning outcomes

## Abstract

**Objective:**

To evaluate the efficacy of a hybrid teaching model integrating the Flipped Classroom (FC) and Case-Based Learning (CBL) in undergraduate *Epidemiology* education, and to compare its outcomes with standalone CBL and traditional Lecture-Based Learning (LBL), thereby providing theoretical evidence for curricular reform.

**Methods:**

A quasi-experimental design was employed involving students majoring in Preventive Medicine (*n* = 100) and Clinical Medicine (*n* = 98) at a university in Yunnan from January 2023 to December 2023. Participants were stratified into three groups: the Experimental Group (Preventive Medicine; 20% Flipped Classroom + 40% Case-Based Learning + 40% Lecture-Based Learning), the Internal Control Group (Preventive Medicine; 40% Case-Based Learning + 60% Lecture-Based Learning), and the External Control Group (Clinical Medicine; 100% Lecture-Based Learning). Outcome measures included competency enhancement, self-reported satisfaction, overall course evaluation, and knowledge acquisition assessed via pre- and post-course assessments.

**Results:**

Significant differences were observed across the three groups regarding competency enhancement, specifically in the breadth of knowledge mastery (*Z* = 8.871, *p* = 0.012), learning interest (*Z* = 11.999, *p* = 0.002), self-directed learning agency (*Z* = 6.736, *p* = 0.034), critical thinking (*Z* = 7.511, *p* = 0.023), literature retrieval skills (*Z* = 6.771, *p* = 0.034), and teamwork capabilities (*Z* = 10.938, *p* = 0.004). In terms of satisfaction, significant variations were found in perceived overall learning effectiveness (*Z* = 13.154, *p* = 0.001), instructional methods (*Z* = 8.679, *p* = 0.013), instructional design (*Z* = 10.920, *p* = 0.004), classroom atmosphere (*Z* = 6.494, *p* = 0.039), and engagement (*Z* = 6.555, *p* = 0.038). Regarding overall course evaluation, the hybrid model showed significant advantages in facilitating academic writing proficiency (*Z* = 9.722, *p* = 0.008), professional technical skills (*Z* = 19.378, *p* < 0.001), and preparation for advanced studies (*Z* = 17.776, *p* < 0.001). Post-test scores differed significantly among the groups (*p* < 0.001), with the Experimental Group achieving the highest mean score (68.2 ± 12.0), followed by the Internal Control Group (66.5 ± 10.6), and the External Control Group (59.6 ± 10.3).

**Conclusion:**

The hybrid pedagogical model combining the Flipped Classroom and Case-Based Learning effectively enhances learning outcomes in undergraduate *Epidemiology*. It demonstrates distinct advantages in stimulating learning interest, fostering self-directed learning and critical thinking, and improving academic performance compared to traditional methods.

## Introduction

*Epidemiology* serves as a cornerstone course for majors in Public Health and Preventive Medicine. It plays a pivotal role in cultivating students’ abilities to analyze disease distribution and determinants, fostering scientific research thinking, and enhancing evidence-based decision-making capabilities (1–3). However, traditional Lecture-Based Learning (LBL), characterized by a teacher-centered approach and didactic instruction, often prioritizes knowledge transmission over active student engagement (4, 5). Consequently, this model often falls short in fostering the critical thinking, problem-solving abilities, and practical competencies required for modern medical practice. In recent years, student-centered pedagogies have garnered increasing attention. Among these, Case-Based Learning (CBL) has been widely adopted in medical education. By integrating theoretical knowledge with practical scenarios through authentic or simulated cases, CBL positions the case as the focal point and the student as the active agent (6, 7). This problem-based, small-group discussion approach is instrumental in enhancing students’ analytical and problem-solving skills (7, 8). Simultaneously, the Flipped Classroom has emerged as an innovative instructional strategy. By shifting knowledge acquisition to the pre-class phase, it reserves in-class time for interactive discourse and collaboration, thereby significantly bolstering student agency and engagement (9, 10). As an “inverted innovation” in instructional organization, the Flipped Classroom transforms the traditional “instruction-then-learning” sequence into “learning-then-instruction.” This structural shift facilitates early knowledge transmission and strengthens knowledge internalization, effectively transitioning students from passive recipients to active learners capable of deep learning and complex problem-solving (11, 12). Despite the documented superiority of both CBL and the Flipped Classroom over traditional LBL, standalone implementations possess inherent limitations. For instance, CBL may lack systematic knowledge structuring, while the efficacy of the Flipped Classroom is heavily contingent upon students’ self-regulated learning abilities and the quality of instructional design (13, 14). Therefore, a Hybrid Teaching Model (or Blended Learning) that synergizes the Flipped Classroom with CBL—supplemented by a proportionate amount of LBL to scaffold the knowledge framework—may achieve a more balanced integration of systematic knowledge acquisition and competency development. Currently, research regarding the integration of the Flipped Classroom and CBL within *Epidemiology* education remains limited. There is a paucity of empirical studies employing multi-group control designs and multidimensional evaluations of teaching effectiveness. Furthermore, existing literature often focuses on isolated metrics, lacking a systematic assessment of learning competencies, satisfaction, and comprehensive literacy. Consequently, this study aims to systematically compare different instructional models through a scientifically designed intervention, providing robust evidence-based insights for the pedagogical reform of *Epidemiology*.

## Methods

### Study participants and setting

A quasi-experimental design was employed for this study. The participants comprised undergraduate students majoring in Preventive Medicine and Clinical Medicine at a university in Yunnan, China, who were enrolled in the *Epidemiology* course between January 2023 and December 2023.

Students from the Preventive Medicine program were allocated into two groups based on the instructional design: the Experimental Group and the Internal Control Group. To ensure comparability in instructional delivery, students from the Clinical Medicine program, who were instructed by the same teaching team, were assigned as the External Control Group. The eligibility criteria were as follows:

- *Inclusion criteria*: (1) Currently enrolled undergraduate students in Preventive Medicine or Clinical Medicine participating in the *Epidemiology* course; (2) Voluntary participation in the study with the completion of all required surveys and assessments.
- *Exclusion criteria*: (1) Students who did not complete the full instructional process (e.g., due to absence); (2) Participants who submitted incomplete questionnaires or responses with logical inconsistencies.

As this study was conducted within the context of educational reform practice, participation was strictly voluntary and anonymous. The research protocol ensured that no personal privacy or sensitive information was compromised.

### Instructional design

#### Experimental group

This group implemented a hybrid teaching model integrating the Flipped Classroom, Case-Based Learning (CBL), and Lecture-Based Learning (LBL), with a proportional distribution of 20, 40, and 40%, respectively. Prior to class, students were organized into small groups of 3–4 members, with a designated group leader responsible for coordinating learning activities and discussions. The instructional process involved students engaging in self-directed learning and conducting group discussions and presentations centered on classic cases, followed by the instructor providing guidance and addressing queries.

#### Internal control group

This group employed a teaching model where CBL served as a precursor to LBL, comprising 40% CBL and 60% LBL. In this setting, the “case analysis” acted as a guiding process where instructors facilitated problem-oriented discussions. Subsequently, didactic lectures were delivered to provide a systematic explanation of key concepts and difficult topics.

#### External control group

This group received traditional Lecture-Based Learning (LBL) exclusively (100%). Instruction was delivered by the instructor in strict accordance with the teaching syllabus, with students primarily assuming the role of passive listeners.

**Figure 1:**
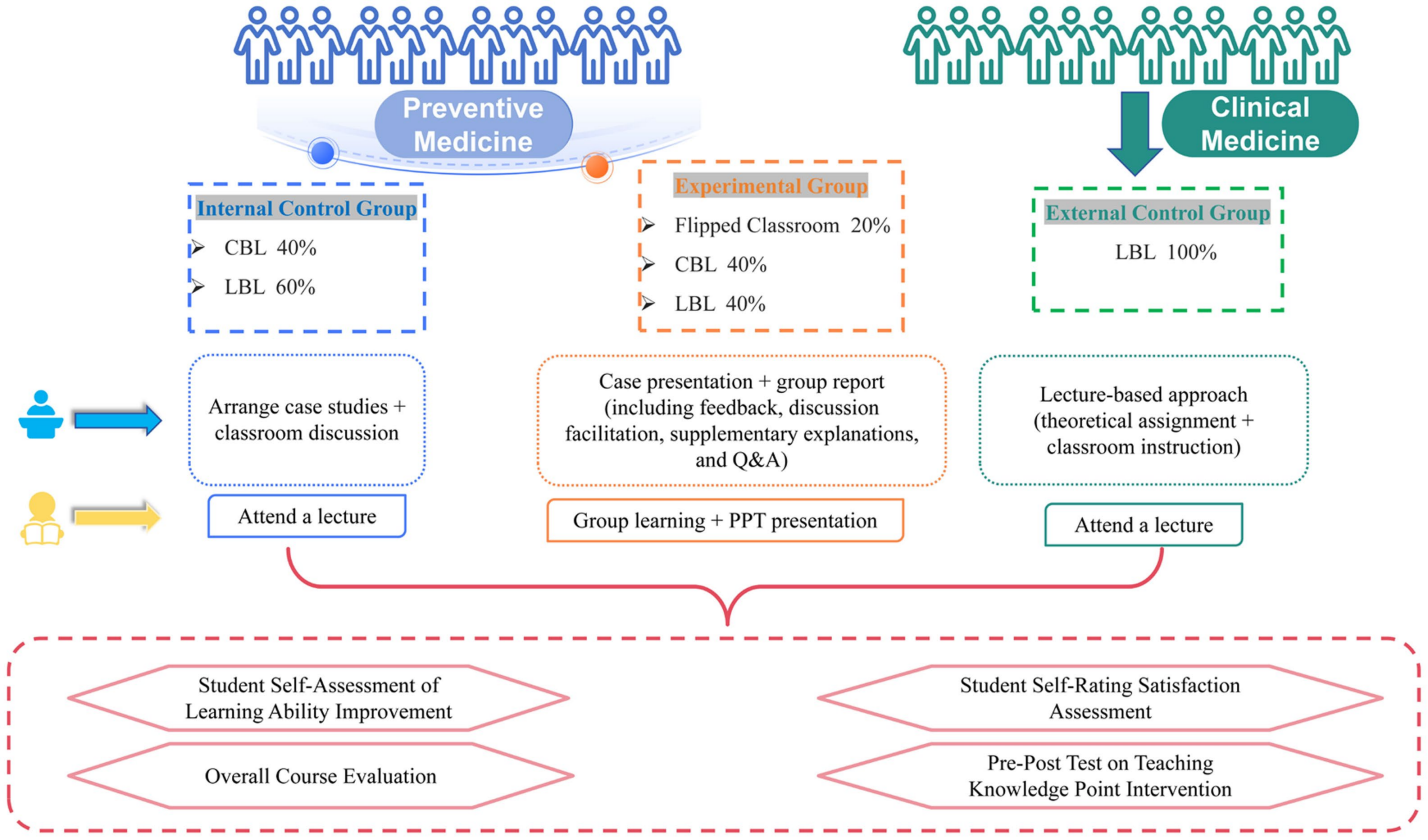
Teaching design flowchart.

The specific instructional workflow is illustrated in Figure 1.

### Outcome measures

The study evaluated the intervention across four dimensions: competency enhancement, student satisfaction, overall course evaluation, and knowledge acquisition.

#### Competency enhancement

This dimension assessed a wide range of indicators, including the breadth and depth of knowledge mastery, retention and understanding of content, identification of key concepts, analytical and problem-solving skills, learning interest, self-directed learning agency, classroom atmosphere, information literacy (retrieval and reading), verbal communication, teamwork, and logical thinking. Perceptions of course difficulty and acceptability were also measured. Each indicator was rated on a 5-point scale ranging from “Excellent” to “Fail” (or “Very Poor”).

#### Student satisfaction

Self-reported satisfaction was evaluated based on indicators such as learning outcomes, improvement in self-directed learning, instructional methods, and academic performance. Responses were recorded on a 5-point Likert scale ranging from “Very Satisfied” to “Very Dissatisfied.”

#### Overall course evaluation

Students provided a holistic evaluation of the course, focusing on overall difficulty, acceptability, cultivation of research thinking, assistance with academic writing, development of professional skills, and preparation for advanced studies. These items were rated on a 5-point scale ranging from “Very Helpful” to “Not Helpful at All.”

#### Knowledge acquisition

Knowledge mastery was objectively assessed using standardized pre-course and post-course tests to evaluate the students’ grasp of epidemiological concepts.

### Statistical analysis

Statistical analyses were performed using R software (version 4.5.1). Categorical data were presented as frequencies and percentages [*n* (%)]. Non-parametric tests were employed for the analysis of ordinal data. A *P* -value of less than 0.05 (*p* < 0.05) was considered statistically significant.

## Results

### Comparison of baseline characteristics

A comparison of pre-test scores revealed no statistically significant differences among the Experimental Group (64.5 ± 11.3), the Internal Control Group (65.1 ± 11.8), and the External Control Group (64.1 ± 10.9) (*F* = 1.475, *p* = 0.232). This indicates that the baseline academic proficiency was comparable across the three groups.

### Impact of different instructional models on competency enhancement

The analysis revealed statistically significant differences among the three groups regarding the breadth of knowledge mastery (*Z* = 8.871, *p* = 0.012). Specifically, the proportion of students rating the instruction as “Very Helpful” was higher in both the Experimental and Internal Control groups compared to the External Control group.

Significant inter-group differences were also observed in learning interest (*Z* = 11.999, *p* = 0.002), where the combined proportions of “Very Helpful” and “Helpful” were superior in the Experimental and Internal Control groups relative to the External Control group. Furthermore, statistically significant differences were found across the groups in self-directed learning agency (*Z* = 6.736, *p* = 0.034), critical thinking skills (*Z* = 7.511, *p* = 0.023), literature retrieval skills (*Z* = 6.771, *p* = 0.034), and teamwork and communication skills (*Z* = 10.938, *p* = 0.004). For the latter four indicators, the Experimental and Internal Control groups demonstrated higher proportions of positive ratings (“Very Helpful” and “Helpful”) than the External Control group. Pairwise comparisons indicated a graded trend for breadth of knowledge mastery, learning interest, and self-directed learning agency, following the order: Experimental Group > Internal Control Group > External Control Group. Detailed data are presented in Tables 1, 2.

**Table 1:** Impact of different instructional models on knowledge mastery.

Item	Group	Very helpful	Helpful	Neutral	Not helpful	Not helpful at all	*Z*	*p*
Breadth of knowledge mastery	Exp.	4 (7.7)	38 (25.3)	9 (6.0)	1 (0.7)	0 (0.0)	8.871	0.012
	Int. Ctrl#	11 (7.3)	38 (25.3)	3 (2.0)	0 (0.0)	0 (0.0)		
	Ext. Ctrl*	5 (3.3)	30 (28.3)	11 (7.3)	0 (0.0)	0 (0.0)		
Depth of knowledge mastery	Exp.	3 (2.0)	33 (22.0)	15 (10)	1 (0.7)	0 (0.0)	5.176	0.075
	Int. Ctrl	11 (7.3)	30 (20.0)	11 (7.3)	0 (0.0)	0 (0.0)		
	Ext. Ctrl	8 (5.3)	29 (19.3)	9 (6.0)	0 (0.0)	0 (0.0)		
Memory and understanding	Exp.	5 (3.3)	33 (22.0)	12 (8.0)	2 (1.3)	0 (0.0)	4.785	0.091
	Int. Ctrl	12 (8.0)	32 (21.3)	7 (4.7)	1 (0.7)	0 (0.0)		
	Ext. Ctrl	6 (4.0)	28 (18.7)	11 (7.3)	1 (0.7)	0 (0.0)		
Grasping key points	Exp.	6 (4.0)	37 (24.7)	6 (4.0)	3 (2.0)	0 (0.0)	1.468	0.48
	Int. Ctrl	13 (8.7)	30 (20.0)	7 (4.7)	2 (1.3)	0 (0.0)		
	Ext. Ctrl	7 (4.7)	31 (20.7)	8 (5.3)	0 (0.0)	0 (0.0)		
Clarity of learning objectives	Exp.	9 (6.0)	32 (21.3)	9 (6.0)	2 (1.3)	0 (0.0)	3.368	0.186
	Int. Ctrl	16 (10.7)	27 (18.0)	9 (6.0)	0 (0.0)	0 (0.0)		
	Ext. Ctrl	11 (7.3)	19 (12.7)	15 (10.0)	1 (0.7)	0 (0.0)		

^#^*p* < 0.05 compared with the Experimental Group; *p < 0.05 compared with the Internal Control Group.

**Table 2:** Impact of different instructional models on competency development.

Item	Group	Very helpful	Helpful	Neutral	Not helpful	Not helpful at all	*Z*	*p*
Learning interest	Exp.	8 (5.3)	29 (19.3)	12 (8.0)	1 (0.7)	2 (1.3)	11.999	0.002
	Int. Ctrl#	19 (12.7)	27 (18.0)	5 (3.3)	1 (0.7)	0 (0.0)		
	Ext. Ctrl*	8 (5.3)	21 (14.0)	14 (9.3)	3 (2.0)	0 (0.0)		
Self-directed learning agency	Exp.	12 (8.0)	29 (19.3)	8 (5.3)	1 (0.7)	2 (1.3)	6.736	0.034
	Int. Ctrl#	22 (14.7)	24 (16.0)	6 (4.0)	0 (0.0)	0 (0.0)		
	Ext. Ctrl*	11 (7.3)	24 (16.0)	9 (6.0)	2 (1.3)	0 (0.0)		
Problem-solving skills	Exp.	13 (8.7)	29 (19.3)	7 (4.7)	2 (1.3)	2 (1.3)	5.269	0.072
	Int. Ctrl	18	32 (21.3)	2 (1.3)	0 (0.0)	0 (0.0)		
	Ext. Ctrl	13 (8.7)	24 (16.0)	9 (6.0)	0 (0.0)	0 (0.0)		
Critical thinking skills	Exp.	10 (6.7)	31 (20.7)	8 (5.3)	1 (0.7)	2 (1.3)	7.511	0.023
	Int. Ctrl	21 (14.0)	25 (16.7)	6 (4.0)	0 (0.0)	0 (0.0)		
	Ext. Ctrl	9 (6.0)	8 (5.3)	8 (5.3)	1 (0.7)	0 (0.0)		
Literature retrieval skills	Exp.	13 (8.7)	28 (18.7)	10 (6.7)	0 (0.0)	1 (0.7)	6.771	0.034
	Int. Ctrl	16 (10.7)	28 (18.7)	7 (4.7)	1 (0.7)	0 (0.0)		
	Ext. Ctrl	8 (5.3)	20 (13.3)	17 (11.3)	1 (0.7)	0 (0.0)		
Literature reading skills	Exp.	11 (7.3)	27 (18.0)	12 (8.0)	1 (0.7)	1 (0.7)	2.192	0.334
	Int. Ctrl	13 (8.7)	28 (18.7)	10 (6.7)	1 (0.7)	0 (0.0)		
	Ext. Ctrl	8 (5.3)	22 (14.7)	14 (9.3)	2 (1.3)	0 (0.0)		
Verbal expression skills	Exp.	20 (13.3)	25 (16.7)	6 (4.0)	1 (0.7)	0 (0.0)	4.238	0.12
	Int. Ctrl	21 (14.0)	27 (18.0)	4 (2.7)	0 (0.0)	0 (0.0)		
	Ext. Ctrl	12 (8.0)	24 (16.0)	10 (6.7)	0 (0.0)	0 (0.0)		
Teamwork & communication	Exp.	21 (14.0)	29 (19.3)	2 (1.3)	0 (0.0)	0 (0.0)	10.938	0.004
	Int. Ctrl	24 (16.0)	26 (17.3)	2 (1.3)	0 (0.0)	0 (0.0)		
	Ext. Ctrl	11 (7.3)	24 (16.0)	11 (7.3)	0 (0.0)	0 (0.0)		

^#^*p* < 0.05 compared with the Experimental Group; *p < 0.05 compared with the Internal Control Group.

### Impact of different instructional models on student satisfaction

Statistically significant differences were observed among the three groups across all dimensions of student satisfaction. These included overall learning effectiveness (*Z* = 13.154, *p* = 0.001), instructional methods (*Z* = 8.679, *p* = 0.013), instructional design (*Z* = 10.920, *p* = 0.004), classroom atmosphere (*Z* = 6.494, *p* = 0.039), and classroom engagement (*Z* = 6.555, *p* = 0.038). Pairwise comparisons revealed a consistent hierarchical trend across these metrics, with the Experimental Group reporting the highest satisfaction, followed by the Internal Control Group, and the External Control Group reporting the lowest. Detailed results are presented in Table 3.

**Table 3:** Comparison of student self-reported satisfaction across different instructional methods.

Item	Group	Very satisfied	Satisfied	Neutral	Dissatisfied	Very dissatisfied	*Z*	*p*
Overall learning effectiveness	Exp.	1 (0.7)	38 (25.3)	13 (8.7)	0 (0.0)	0 (0.0)	13.154	0.001
	Int. Ctrl#	14 (9.3)	33 (22.0)	5 (3.3)	0 (0.0)	0 (0.0)		
	Ext. Ctrl*	6 (4.0)	31 (20.7)	9 (6.0)	0 (0.0)	0 (0.0)		
Improvement in self-directed learning	Exp.	13 (8.7)	25 (16.7)	14 (9.3)	0 (0.0)	0 (0.0)	0.205	0.902
	Int. Ctrl	9 (6.0)	36 (24.0)	7 (4.7)	0 (0.0)	0 (0.0)		
	Ext. Ctrl	10 (6.7)	26 (17.3)	10 (6.7)	0 (0.0)	0 (0.0)		
Instructional methods	Exp.	7 (4.7)	32 (21.3)	12 (8.0)	1 (0.7)	0 (0.0)	8.679	0.013
	Int. Ctrl#	19 (12.7)	27 (18.0)	6 (4.0)	0 (0.0)	0 (0.0)		
	Ext. Ctrl*	10 (6.7)	29 (19.3)	7 (4.7)	0 (0.0)	0 (0.0)		
Instructional design	Exp.	9 (6.0)	31 (20.7)	12 (8.0)	0 (0.0)	0 (0.0)	10.92	0.004
	Int. Ctrl#	22 (14.7)	26 (17.3)	4 (2.7)	0 (0.0)	0 (0.0)		
	Ext. Ctrl*	10 (6.7)	28 (18.7)	7 (4.7)	1 (0.7)	0 (0.0)		
Classroom atmosphere	Exp.	14 (9.3)	23 (15.3)	15 (10.0)	0 (0.0)	0 (0.0)	6.494	0.039
	Int. Ctrl#	22 (14.7)	25 (16.7)	5 (3.3)	0 (0.0)	0 (0.0)		
	Ext. Ctrl*	11 (7.3)	31 (20.7)	4 (2.7)	0 (0.0)	0 (0.0)		
Classroom engagement	Exp.	12 (8.0)	31 (20.7)	7 (4.7)	2 (1.3)	0 (0.0)	6.555	0.038
	Int. Ctrl#	21 (14.0)	27 (18.0)	4 (2.7)	0 (0.0)	0 (0.0)		
	Ext. Ctrl*	10 (6.7)	28 (18.7)	7 (4.7)	1 (0.7)	0 (0.0)		

^#^*p* < 0.05 compared with the Experimental Group; *p < 0.05 compared with the Internal Control Group.

### Impact of different instructional models on overall course evaluation

The study revealed significant differences among the three groups regarding the course’s perceived utility in academic writing (*Z* = 9.722, *p* = 0.008), the cultivation of professional technical skills (*Z* = 19.378, *p* < 0.001), and preparation for advanced studies (*Z* = 17.776, *p* < 0.001). Pairwise comparisons demonstrated a consistent trend where the Experimental Group rated the course significantly higher than the Internal Control Group, which in turn rated it higher than the External Control Group. Detailed data are provided in Table 4.

**Table 4:** Comparison of overall course evaluation across different instructional methods.

Item	Group	Very helpful	Helpful	Neutral	Not helpful	Not helpful at all	*Z*	*P*
Overall course difficulty	Exp.	2 (1.3)	24 (16.0)	20 (13.3)	6 (4.0)	0 (0.0)	0.374	0.83
	Int. Ctrl	0 (0.0)	29 (19.3)	20 (13.3)	3 (2.0)	0 (0.0)		
	Ext. Ctrl	3 (2.0)	20 (13.3)	21 (14.0)	2 (1.3)	0 (0.0)		
Overall course acceptability	Exp.	3 (2.0)	43 (28.7)	6 (4.0)	0 (0.0)	0 (0.0)	1.189	0.552
	Int. Ctrl	4 (7.7)	45 (37.2)	3 (2.0)	0 (0.0)	0 (0.0)		
	Ext. Ctrl	5 (3.3)	33 (22.0)	8 (5.3)	0 (0.0)	0 (0.0)		
Cultivation of research thinking	Exp.	13 (8.7)	33 (22.0)	6 (4.0)	0 (0.0)	0 (0.0)	5.164	0.076
	Int. Ctrl	13 (8.7)	31 (20.7)	7 (4.7)	1 (0.7)	0 (0.0)		
	Ext. Ctrl	5 (3.3)	30 (20.0)	11 (7.3)	0 (0.0)	0 (0.0)		
Help with academic writing	Exp.	15 (10.0)	23 (15.3)	14 (9.3)	0 (0.0)	0 (0.0)	9.722	0.008
	Int. Ctrl#	19 (12.7)	24 (16.0)	8 (5.3)	1 (0.7)	0 (0.0)		
	Ext. Ctrl*	5 (3.3)	24 (16.0)	15 (10.0)	1 (0.7)	1 (0.7)		
Cultivation of professional skills	Exp.	17 (11.3)	28 (18.7)	7 (4.7)	0 (0.0)	0 (0.0)	19.378	<0.001
	Int. Ctrl#	26 (17.3)	22 (14.7)	3 (2.0)	1 (0.7)	0 (0.0)		
	Ext. Ctrl*	5 (3.3)	28 (18.7)	12 (8.0)	1 (0.7)	0 (0.0)		
Preparation for advanced studies	Exp.	13 (8.7)	32 (21.3)	7 (4.7)	0 (0.0)	0 (0.0)	17.776	<0.001
	Int. Ctrl#	25 (16.7)	25 (16.7)	1 (0.7)	1 (0.7)	0 (0.0)		
	Ext. Ctrl*	7 (4.7)	27 (18.0)	11 (7.3)	1 (0.7)	0 (0.0)		

^#^*p* < 0.05 compared with the Experimental Group; *p < 0.05 compared with the Internal Control Group.

### Impact of different instructional models on academic performance

Post-course assessment scores showed statistically significant differences among the three groups (*F* = 8.318, *p* < 0.001). Pairwise comparisons indicated that the Experimental Group achieved significantly higher scores than the Internal Control Group, and the Internal Control Group scored significantly higher than the External Control Group (*p* < 0.05). See Table 5 for details.

**Table 5:** Comparison of post-course assessment scores across different groups.

Assessment score	Experimental group	Internal control group#	External control group*	*F*	*p*
Post-course	68.2 ± 12.0	66.5 ± 10.6	59.6 ± 10.3	8.318	<0.001

^#^*p* < 0.05 compared with the Experimental Group; *p < 0.05 compared with the Internal Control Group.

## Discussion

Based on a quasi-experimental design, this study systematically compared the effectiveness of a hybrid Flipped Classroom combined with CBL model, a CBL-LBL combined model, and a traditional LBL model in undergraduate *Epidemiology* education. The results indicate that the hybrid Flipped Classroom and CBL model offers distinct advantages in broadening knowledge mastery, stimulating learning interest, enhancing self-directed learning agency, and improving overall knowledge acquisition. These findings suggest that this hybrid model holds significant value for pedagogical reform in *Epidemiology*.

Regarding learning interest and self-directed learning capabilities, the Experimental Group outperformed the Internal Control Group, which in turn performed better than the External Control Group. This outcome may be attributed to the instructional structure of the Flipped Classroom, which emphasizes pre-class self-study and in-depth in-class participation (10, 14, 15). The course design fully leveraged MOOC platforms and online resources to cover the entire instructional process, offering diverse and engaging learning formats. By forming a preliminary cognitive framework through pre-class preparation and subsequently deepening their understanding through discussions of practical cases, students were able to enhance their learning motivation and initiative (14, 16, 17). Previous studies have similarly indicated that the Flipped Classroom significantly boosts student engagement and involvement, facilitating a transition from passive reception to active acquisition (12, 18). In terms of literature retrieval and research literacy, both the Experimental and Internal Control groups outperformed the External Control group. CBL guides students to identify problems, retrieve literature, analyze issues, and propose solutions through authentic cases. This process necessitates the consultation of extensive literature. Through mutual discussion and debate, students can deepen the breadth and depth of their learning, which fosters critical reflection, enhances problem-solving abilities, and strengthens information retrieval skills and evidence-based thinking (19–21). Furthermore, both the Flipped Classroom and CBL emphasize collaborative learning. Through division of labor, discussion, and presentation of outcomes, these methods enhance students’ communication skills and team awareness. Relevant studies indicate that collaborative learning significantly improves students’ social interaction skills and learning outcomes (22, 23).

Regarding academic performance, the Experimental Group outperformed the Internal Control Group, which in turn performed better than the External Control Group. This finding suggests that the hybrid Flipped Classroom and CBL model not only enhances subjective learning experiences but also improves objective learning outcomes (24, 25). The underlying mechanism may be that the Flipped Classroom improves the efficiency of in-class time utilization by shifting knowledge transmission to the pre-class phase, while CBL strengthens knowledge transfer and application through situated learning. Together, they form a “closed-loop” process of “knowledge transmission → knowledge absorption → knowledge enhancement.”

These results further validate the advantages of the hybrid model in improving learning effectiveness. The potential mechanisms include (21, 25, 26):

(1) The Flipped Classroom creates a repeated learning effect through pre-class preview and in-class review, which facilitates knowledge consolidation.
(2) CBL promotes deep processing of knowledge through problem analysis, thereby enhancing learning outcomes.
(3) The shift from passive reception to active participation improves learning efficiency and the mastery of knowledge.

Regarding student self-reported satisfaction, statistically significant differences were observed among the three groups in indicators such as overall learning effectiveness, instructional methods, instructional design, classroom atmosphere, and engagement, with the Experimental Group reporting the highest satisfaction. These findings suggest that the hybrid teaching model offers distinct advantages in optimizing the learning experience. First, the Flipped Classroom combines pre-class learning with in-class interaction, transforming the classroom from a setting of unilateral didactic lecturing to one of multidirectional interaction between instructors and students, thereby significantly improving the classroom atmosphere and student engagement (24, 27). Second, CBL enhances the situated nature of instruction through the introduction of cases, enabling students to integrate theoretical knowledge with practical problems, thus increasing the utility and interest of learning (8, 16). Notably, the Experimental Group demonstrated particular advantages in classroom engagement and atmosphere, which may be attributed to its more flexible and highly interactive instructional structure. Existing research indicates that high-interaction classroom environments significantly enhance student satisfaction and learning investment (28, 29).

In terms of overall course evaluation, the Experimental Group performed significantly better than the other two groups regarding academic writing skills, professional technical skills, and preparation for advanced studies. This result indicates that the hybrid model not only improves short-term learning outcomes but also exerts a positive impact on students’ long-term academic development. The potential reasons include (30, 31):

(1) The flipped classroom requires students to retrieve and synthesize literature for presentation before class, which helps improve research literacy and academic writing skills.
(2) CBL promotes the integration of theory and practice through case analysis, thereby enhancing professional technical skills.
(3) The hybrid model strengthens knowledge understanding and application, fostering continuous learning capabilities that support further academic pursuits.

### Limitations

Several limitations should be acknowledged. First, some evaluation indicators in this study were derived from student self-reported questionnaires, which are inherently subjective and may introduce information bias. Although a multidimensional evaluation system was employed, the study lacked more objective assessment tools. Future research plans to incorporate or construct standardized assessment systems. Second, this study focused on short-term effects immediately following the course and did not include a follow-up study on students’ long-term learning outcomes.

## Conclusion

In summary, the entire instructional process was underpinned by the philosophy of Outcome-Based Education (OBE), placing the student at the center. Under the hybrid teaching model integrating the Flipped Classroom and Case-Based Learning, students demonstrated improved self-perceived learning competencies and higher satisfaction levels. Furthermore, their practical and research skills were effectively exercised. This model plays a positive role in fostering research capabilities and professional skills while enhancing interaction between students and instructors and supporting personalized learning. Consequently, this study provides a theoretical reference for constructing a novel hybrid teaching model for the *Epidemiology* curriculum.

## Data Availability

The original contributions presented in the study are included in the article/supplementary material, further inquiries can be directed to the corresponding authors.
